# Site-specific biomechanical alterations of the knee during gait in ACL-deficient patients with concomitant cartilage lesions

**DOI:** 10.3389/fbioe.2026.1800182

**Published:** 2026-05-18

**Authors:** Shilin Li, Wen Liu, Xin Xiao, Wei Zhou, Yueming Gu, Jie Zhou, Mingfeng Lu, Wei Chen

**Affiliations:** 1 Department of Rehabilitation Therapy Teaching and Research, Gannan Health Vocational College, Ganzhou, China; 2 The Eighth Clinical Medical College of Guangzhou University of Chinese Medicine, Guangzhou University of Chinese Medicine, Foshan, China; 3 Foshan Hospital of Traditional Chinese Medicine, Guangzhou University of Chinese Medicine, Foshan, China; 4 Xiang Xing College of Hunan University of Traditional Chinese Medicine, Hunan University of Chinese Medicine, Changsha, China; 5 School of Rehabilitation Medicine, Gannan Medical University, Ganzhou, China

**Keywords:** anterior cruciate ligament, cartilage, kinematics, knee, walking

## Abstract

**Background:**

Anterior cruciate ligament (ACL) deficiency often coexists with articular cartilage damage, yet the biomechanical effects of lesion location on knee kinematics during gait remain unclear. This study investigated the association between cartilage lesion site and six degrees of freedom (6-DOF) knee kinematics in ACL-deficient knees.

**Methods:**

A total of 116 patients with unilateral ACL deficiency were divided into four groups: isolated ACL deficiency (ACLDI), ACL with femoral cartilage lesion (ACLDF), ACL with tibial cartilage lesion (ACLDT), and ACL with combined femoral–tibial lesions (ACLDC). Three-dimensional motion capture was used to record knee 6-DOF kinematics during walking. One-way ANOVA and Statistical Parametric Mapping (SPM1d) were applied to identify intergroup differences. Key discrete gait parameters were further reanalyzed using meniscal-adjusted linear models.

**Results:**

Meniscal-adjusted analyses indicated that group differences remained in selected gait parameters. Specifically, differences were identified in the abduction–adduction, flexion–extension, and anterior–posterior displacement dimensions. Compared with the ACLDI group, ACLDT and ACLDC patients exhibited greater adduction and anterior tibial translation during the stance phase, while ACLDF and ACLDC showed reduced knee flexion and overall range of motion.

**Conclusion:**

In ACL-deficient knees, gait biomechanical alterations are associated with cartilage lesion location. Different lesion locations appear to correspond to distinct functional phenotypes, suggesting that lesion-specific assessment may improve identification of residual instability and inform more individualized rehabilitation strategies to restore joint stability and reduce the risk of secondary degeneration.

## 1 Background

Anterior cruciate ligament (ACL) injury is one of the most common knee injuries in physically active populations and is frequently accompanied by damage to other intra-articular structures, particularly the articular cartilage (Lohmander et al., 2007; Øiestad et al., 2009; Røtterud et al., 2011). In ACL-deficient (ACLD)knees, concomitant cartilage lesions are clinically relevant because they are associated with worse joint function, persistent symptoms, and an elevated risk of post-traumatic osteoarthritis (Culvenor et al., 2019; Louboutin et al., 2009). Accordingly, the functional consequences of ACL deficiency should not be interpreted solely as a problem of ligament insufficiency, but rather as the result of combined structural disturbances within the joint (Lohmander et al., 2007; Øiestad et al., 2009; Ajuied et al., 2014; Luc et al., 2014).

Following ACL rupture, abnormal tibiofemoral motion alters the mechanical environment of the knee (Andriacchi et al., 2009; Chaudhari et al., 2008; Li et al., 2006). Excessive anterior tibial translation, rotational instability, and disturbed load distribution may increase local contact stress and shear forces on the articular surfaces, thereby contributing to cartilage degeneration and progressive joint dysfunction (Stergiou et al., 2007; Orsi et al., 2016; Andriacchi and Mündermann, 2006). However, cartilage damage in the ACLD knee should not be regarded as a homogeneous condition. Because the femoral condyles and tibial plateau play distinct roles in load transmission, articular congruity, and the rolling–sliding mechanism of knee motion, lesions occurring at different anatomical sites may have different biomechanical consequences during dynamic activities such as walking (Andriacchi et al., 2009; Chaudhari et al., 2008; Li et al., 2006; Stergiou et al., 2007; Orsi et al., 2016; Andriacchi and Mündermann, 2006).

This distinction may be particularly important for understanding functional gait adaptations. Because femoral and tibial cartilage surfaces contribute differently to articular congruity and load transmission, lesions at these sites may be associated with different alterations in sagittal-plane motion, frontal-plane alignment, and anterior-posterior stability during walking. When both surfaces are involved, these biomechanical disturbances may be more pronounced.

Although gait alterations in ACLD knees have been widely investigated, most previous studies have focused on isolated ACL deficiency or concomitant meniscal pathology, rather than cartilage involvement (Hart et al., 2016; Kaur et al., 2016; Slater et al., 2017). Existing studies on cartilage lesions have mainly emphasized lesion prevalence, structural degeneration, or general clinical outcomes, while their dynamic biomechanical effects during functional activities remain insufficiently characterized (Røtterud et al., 2011; Culvenor et al., 2019; Louboutin et al., 2009). In addition, cartilage damage has usually been treated as a single homogeneous entity, with little attention to whether femoral, tibial, and combined femoral–tibial lesions produce distinct 6-DOF kinematic adaptations during walking (Røtterud et al., 2011; Culvenor et al., 2019; Louboutin et al., 2009; Hart et al., 2016; Kaur et al., 2016; Slater et al., 2017).

Therefore, this study compared 6-DOF knee kinematics during gait among patients with isolated ACL deficiency and those with concomitant femoral, tibial, or combined femoral–tibial cartilage lesions. We hypothesized that cartilage lesion location would be associated with distinct gait alterations, particularly in anterior-posterior stability, frontal-plane control, and flexion-extension motion. Clarifying these location-specific biomechanical features may improve biomechanical phenotyping and individualized management in ACLD knees.

## 2 Methods

### 2.1 Participants

**FIGURE 1:**
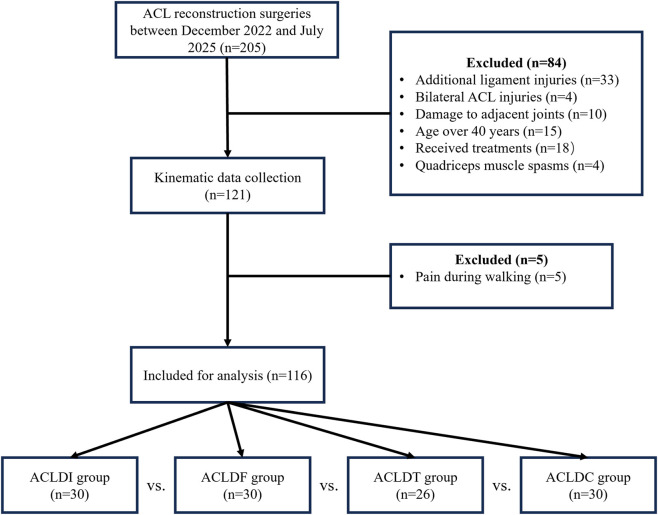
Patient flowchart throughout the study.

A total of 116 patients with unilateral ACLD knees (59 males and 57 females; age range, 20–35 years; body mass index (BMI), 17.6–26.5 kg/m^2^) were recruited for this study. Among them, 30 patients had isolated ACL deficiency (ACLDI), 30 had combined ACL and femoral cartilage injuries (ACLDF), 26 had combined ACL and tibial cartilage injuries (ACLDT), and 30 had combined ACL and both femoral and tibial cartilage injuries (ACLDC). Flowchart of participant enrollment and inclusion process are showed in Figure 1. Detailed demographic comparisons are presented in Table 1. The study protocol was approved by the institutional ethics committee (Ethics Committee of Guangdong Provincial Hospital of Chinese Medicine: ZE 2024–101–01) and conducted in accordance with the Declaration of Helsinki.

**TABLE 1:** Baseline demographic, clinical, and lesion characteristics of the four groups.

Characteristic	ACLDI (n = 30)	ACLDF (n = 30)	ACLDT (n = 26)	ACLDC (n = 30)	*p* value
Sex, n (%)	​	​	​	​	0.84
Male	16 (53.3)	15 (50.0)	14 (53.8)	14 (46.7)	​
Female	14 (46.7)	15 (50.0)	12 (46.2)	16 (53.3)	​
Age, years	27.8 ± 1.7	29.6 ± 2.1	28.3 ± 2.3	27.9 ± 1.9	0.78
Time from injury to operation, months	3.5 ± 1.9	3.6 ± 2.6	3.8 ± 1.5	4.0 ± 1.1	0.08
Meniscal management, n (%)	​	​	​	​	0.94
Partial meniscectomy	12 (40.0)	11 (36.7)	9 (34.6)	10 (33.3)	​
Meniscal repair	12 (40.0)	10 (33.3)	8 (30.8)	11 (36.7)	​
No meniscal procedure	6 (20.0)	9 (30.0)	9 (34.6)	9 (30.0)	​
Cartilage lesion subregions, n (%)	​	​	​	​	<0.001
Medial tibial plateau	0 (0.0)	0 (0.0)	12 (46.2)	14 (46.7)	​
Lateral tibial plateau	0 (0.0)	0 (0.0)	15 (57.7)	15 (53.3)	​
Medial femoral condyle	0 (0.0)	12 (40.0)	0 (0.0)	12 (40.0)	​
Lateral femoral condyle	0 (0.0)	14 (46.7)	0 (0.0)	15 (50.0)	​
Trochlea	0 (0.0)	4 (13.3)	0 (0.0)	3 (10.0)	​
Self-selected treadmill speed, km/h	2.98 ± 0.12	3.05 ± 0.11	2.93 ± 0.10	3.01 ± 0.12	0.18
ICRS grade, n (%)	​	​	​	​	0.62
Grade III	0 (0.0)	14 (46.7)	13 (50.0)	12 (40.0)	​
Grade IV	0 (0.0)	16 (53.3)	13 (50.0)	18 (60.0)	​
Mean total defect size, cm^2^	0	3.1 ± 1.6	2.6 ± 1.5	2.8 ± 1.9	0.39
IKDC subjective score	58.9 ± 10.8	61.9 ± 8.8	59.6 ± 7.1	60.3 ± 8.6	0.45
Tegner activity score	2.7 ± 1.2	2.6 ± 0.6	2.8 ± 1.1	2.8 ± 0.9	0.71
Cause of injury					0.96
Sports injury	18 (60.0)	20 (66.7)	17 (65.4)	16 (53.3)	​
Traffic accident	3 (10.0)	2 (6.7)	1 (3.8)	2 (6.7)	​
Daily activity injury	5 (16.7)	4 (13.3)	6 (23.1)	7 (23.3)	​
Work-related injury	4 (13.3)	4 (13.3)	2 (7.7)	5 (16.7)	​

ACLDI, isolated ACL, deficiency; ACLDF, ACL, deficiency with concomitant femoral cartilage lesion; ACLDT, ACL, deficiency with concomitant tibial cartilage lesion; ACLDC, ACL, deficiency with combined femoral and tibial cartilage lesions; ICRS, international cartilage repair society; IKDC, International Knee Documentation Committee. Continuous variables are presented as mean ± standard deviation, and categorical variables as n (%). P values were calculated using one-way ANOVA, for continuous variables and the chi-square test for categorical variables, as appropriate. Because the study groups were defined according to the broad anatomical distribution of cartilage lesions, differences in cartilage lesion subregions across groups were expected.

#### 2.1.1 The inclusion criteria were as follows

Participants aged 18–50 years, of either sex; unilateral complete ACL rupture beyond the acute post-injury phase (>3 months after injury); ability to walk independently and complete gait testing; and BMI <30 kg/m^2^.

#### 2.1.2 The exclusion criteria were as follows

Grade III injury to other ipsilateral knee ligaments; meniscal root tears requiring surgical intervention or a history of extensive meniscectomy; previous fracture around the ipsilateral knee; inflammatory joint disease (e.g., rheumatoid arthritis or gouty arthritis) or radiographic evidence of generalized osteoarthritis (Kellgren–Lawrence grade ≥ II); neuromuscular disorders affecting gait; pregnancy; and inability to complete gait testing because of pain or other causes. Patients with partial meniscectomy or meniscal repair were not excluded.

**FIGURE 2:**
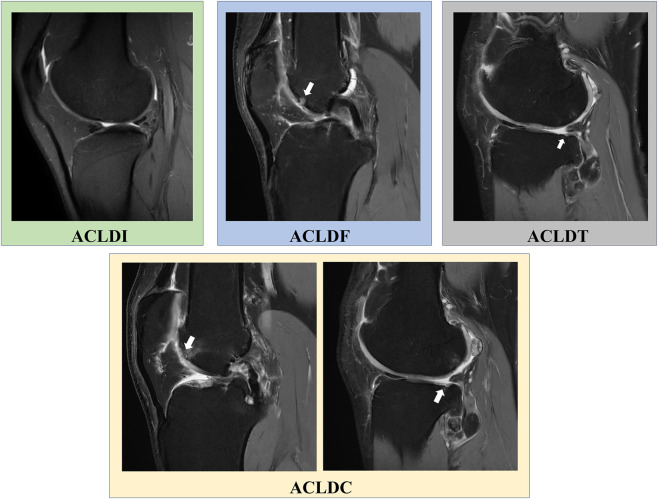
MRI illustrations of the four ACL deficiency groups.

Articular cartilage status was assessed preoperatively by MRI. Patients with concomitant cartilage lesions were required to have focal full-thickness defects graded as International Cartilage Repair Society (ICRS) grade III–IV (Mainil-Varlet et al., 2010), with a lesion area of 1–4 cm^2^. According to lesion location, patients were classified into femoral, tibial, or combined femoral–tibial cartilage lesion groups (Figure 2). In addition to this primary grouping framework, lesion subregions were further documented according to compartmental and anatomical location, including medial versus lateral involvement and the corresponding femoral condyle or tibial plateau regions, as summarized in Table 1.

All gait assessments were performed before primary ACL reconstruction, when patients were in a relatively stable preoperative condition rather than the immediate post-injury phase. Only patients who were able to complete treadmill walking steadily were included. Patients with obvious pain behavior during testing were excluded. Time from injury to operation was recorded as a baseline characteristic.

### 2.2 Gait data collection

#### 2.2.1 Apparatus

Gait data were collected using a navigation-based three-dimensional optical motion capture system (Opti_Knee, Innomotion Inc., Shanghai, China) to record six-degrees-of-freedom (6-DOF) knee kinematic parameters during walking. The system consisted of a binocular infrared stereo tracker (NDI Polaris Spectra, Northern Digital Inc., Canada), a high-speed camera (Basler aca640-90uc, Germany), rigid body markers affixed to the lateral aspects of the femur and tibia, and a digitizing probe for establishing individualized coordinate systems. The sampling frequency was 60 Hz, with a translational accuracy of 0.3 mm root mean square, rotational repeatability better than 1.3°, and translational repeatability better than 0.9 mm (Elfring et al., 2010). Prior to testing, a standardized calibration procedure was performed to ensure measurement consistency across all sessions.

#### 2.2.2 Testing procedures

Before testing, each participant performed 3–5 min of treadmill walking to determine a comfortable walking speed and minimize learning effects. This self-selected treadmill speed was then used for the formal gait assessment and recorded as a baseline characteristic. Subsequently, rigid body markers were attached to the lateral aspects of the femur and tibia on the tested limb. A trained operator used a handheld digitizing probe to identify key bony landmarks—including the medial and lateral malleoli, medial and lateral femoral condyles, greater trochanter, and medial and lateral edges of the tibial plateau—according to standard anatomical definitions for lower-limb motion analysis, and these landmarks were used to establish individualized femoral and tibial coordinate systems. Knee motion was then expressed using a joint coordinate system consistent with the convention of Grood and Suntay (Grood and Suntay, 1983; Zhang et al., 2016).

After coordinate calibration, participants walked continuously on a bidirectional treadmill for approximately 20 s at their self-selected comfortable speed. The system synchronously recorded the 6-DOF knee kinematic trajectories throughout the gait cycle, including flexion–extension, internal–external rotation, adduction–abduction rotation, and translations in the anterior–posterior, medial–lateral, and superior–inferior directions. Each participant completed at least three stable gait cycles, and only trials with complete signals and without marker loss were included for analysis. If marker displacement or abnormal data occurred during testing, recalibration and repeated data acquisition were performed.

All tests were conducted by the same trained technician to minimize operator bias. To ensure data quality, the raw trajectory data were reviewed immediately after acquisition for completeness and then stored as three-dimensional coordinate data for subsequent analysis.

After obtaining the 6-DOF kinematic curves of the knee joint, the gait cycle was segmented into the stance and swing phases according to gait phase division. For each phase (stance, swing, and the entire gait cycle), the maximum value, minimum value, and range of motion (ROM) were calculated for subsequent statistical analysis.

### 2.3 Statistical analysis

All statistical analyses were performed using SPSS version 25.0 (IBM Corp., Armonk, NY, United States) and MATLAB R2021b (MathWorks Inc., Natick, MA, United States). For baseline characteristics, the Shapiro–Wilk test was used to assess the normality of continuous variables. Normally distributed variables were compared among the four groups using one-way analysis of variance (ANOVA), whereas categorical variables were compared using the chi-square test, as appropriate.

For discrete gait parameters derived from each gait phase, including the maximum, minimum, and range of motion, normality was assessed using the Shapiro–Wilk test. Normally distributed data were compared using one-way ANOVA with Bonferroni-adjusted *post hoc* tests, whereas non-normally distributed data were analyzed using the Kruskal–Wallis H test. To address the potential confounding effect of concomitant meniscal treatment, additional covariate-adjusted linear models were constructed for key discrete gait parameters, with group as the main factor and partial meniscectomy, meniscal repair as adjustment variables. Adjusted group means and multiple-comparison-adjusted *post hoc* p values were then estimated from the fitted models. Only parameters showing a significant overall adjusted group effect are presented in Table 2 and Figure 5.

**TABLE 2:** Discrete gait parameters that remained significantly different among groups after adjustment for meniscal variables (Mean ± SD).

Phase	DOF	Para	F	Overall *p*	Effect size (ηp^2^)	ACLDI	ACLDF	ACLDT	ACLDC	*p1*	*p2*	*p3*	*p4*	*p5*	*p6*
Swing	Add-abd	Max	3.06	0.03	0.08	9.3 ± 2.8	3.4 ± 1.5	6.1 ± 3.1	4.1 ± 1.8	0.007	0.144	0.016	0.204	0.764	0.331
Swing	Add-abd	Min	4.82	0.003	0.12	−6.2 ± 4.1	−10.9 ± 4	−10.3 ± 2.5	−16 ± 3.5	0.073	0.115	<0.001	0.827	0.05	0.03
Stance	Flex-ext	Min	2.67	0.05	0.07	−6.2 ± 3	−3.7 ± 2.5	−1.9 ± 3.8	4.6 ± 1.8	0.517	0.271	0.008	0.647	0.041	0.111
Swing	Flex-ext	Max	7.56	<0.001	0.17	59.3 ± 9	54.3 ± 12.2	60.6 ± 17.3	46.3 ± 17.3	0.116	0.792	<0.001	0.066	0.018	<0.001
Swing	Flex-ext	Min	7.78	<0.001	0.18	−0.4 ± 2.8	−12.2 ± 4	−6.1 ± 3.8	−1.5 ± 4.4	<0.001	0.043	0.734	0.025	<0.001	0.09
Swing	Flex-ext	Range	4.51	0.005	0.11	59.7 ± 16.6	66.5 ± 23.6	66.8 ± 28.1	47.8 ± 30.1	0.293	0.282	0.037	0.978	0.002	0.002
Swing	AP	Min	4.97	0.003	0.12	8.2 ± 1.3	8.7 ± 1.7	11.7 ± 1.7	12 ± 2.1	0.78	0.007	0.003	0.015	0.008	0.812

ACLDI, isolated ACL, deficiency; ACLDF, ACL, deficiency with concomitant femoral cartilage lesion; ACLDT, ACL, deficiency with concomitant tibial cartilage lesion; ACLDC, ACL, deficiency with combined femoral and tibial cartilage lesions; DOF, degree of freedom; DOF, abbreviations: Add-Abd, adduction–abduction; Flex-Ext, flexion–extension; AP, anterior–posterior; Values for ACLDI, ACLDF, ACLDT, and ACLDC, are presented as adjusted means, estimated from the covariate-adjusted linear model. The overall adjusted group effect was tested using a linear model with group as the main factor and partial meniscectomy and meniscal repair as adjustment variables; Pairwise p values are multiple-comparison-adjusted *post hoc* p values and are defined as follows: *p1*, ACLDI, vs. ACLDF; *p2*, ACLDI, vs. ACLDT; *p3*, ACLDI, vs. ACLDC; *p4*, ACLDF, vs. ACLDT; *p5*, ACLDF, vs. ACLDC; *p6*, ACLDT, vs. ACLDC., statistically significant adjusted p values are highlighted in red.

For the time-series kinematic curves throughout the entire gait cycle, Statistical Parametric Mapping (SPM1d) analysis was performed (Chang et al., 2016) to identify phase-specific differences among groups during the gait cycle. A one-way SPM-F test was first conducted to assess the overall group effect. When a significant effect was detected, *post hoc* pairwise SPM-t tests were subsequently performed. To account for multiple *post hoc* comparisons, Bonferroni correction was applied according to the number of pairwise group comparisons. All time-series data were normalized to 0%–100% of the gait cycle.

## 3 Results

### 3.1 Baseline characteristics

Detailed baseline demographic, clinical, and lesion characteristics are presented in Table 1. No significant intergroup differences were observed in sex, age, meniscal management, self-selected treadmill speed, ICRS grade, mean total defect size, IKDC subjective score, Tegner activity score, or cause of injury (all p > 0.05). Although the time from injury to operation tended to be longer in the ACLDC group than in the other three groups, the difference did not reach statistical significance (p = 0.08). Detailed lesion localization, including compartmental and anatomical subregions, is summarized in Table 1. As expected, cartilage lesion subregions differed significantly among groups (p < 0.001), because the study groups were defined according to the broad anatomical distribution of cartilage involvement.

### 3.2 Gait interval differences across four groups

**FIGURE 3:**
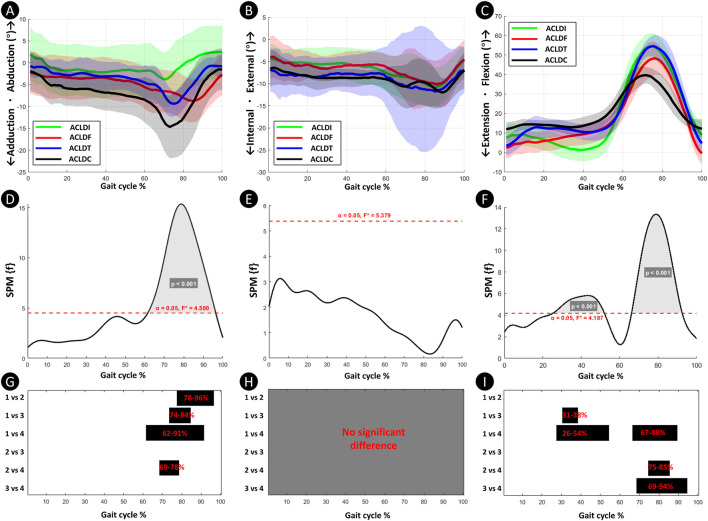
Comparison of knee joint kinematics among the four groups in the adduction–abduction, internal–external rotation, and flexion–extension dimensions throughout the full gait cycle. Plots **(A–C)** Knee joint motion angles for the four groups in the adduction–abduction, internal–external rotation, and flexion–extension dimensions; Plots **(D–F)** One-way SPM-F analyses of intergroup differences across the three dimensions, with shaded regions indicating gait cycle intervals exhibiting statistically significant differences; Plots **(G–I)** Post hoc pairwise SPM-t comparisons between groups for the adduction–abduction, internal–external rotation, and flexion–extension dimensions, where black bars denote gait phases with significant between-group differences. 1 = ACLDI; 2 = ACLDF; 3 = ACLDT; 4 = ACLDC.

Figures 3A–C depict the raw knee joint kinematic curves across four groups during walking, including adduction–abduction, internal–external rotation, and flexion–extension dimensions. SPM-based one-way ANOVA revealed no significant intergroup difference in internal–external rotation throughout the gait cycle. In contrast, significant differences were found in adduction–abduction and flexion–extension dimensions (*p* < 0.001).

As shown in Figures 3G–I, *post hoc* analyses indicated that compared with the ACLDI, ACLDF, ACLDT, and ACLDC groups exhibited significantly greater abduction angles. The ACLDC group also demonstrated more pronounced abduction than ACLDF (*p* < 0.001).

**FIGURE 4:**
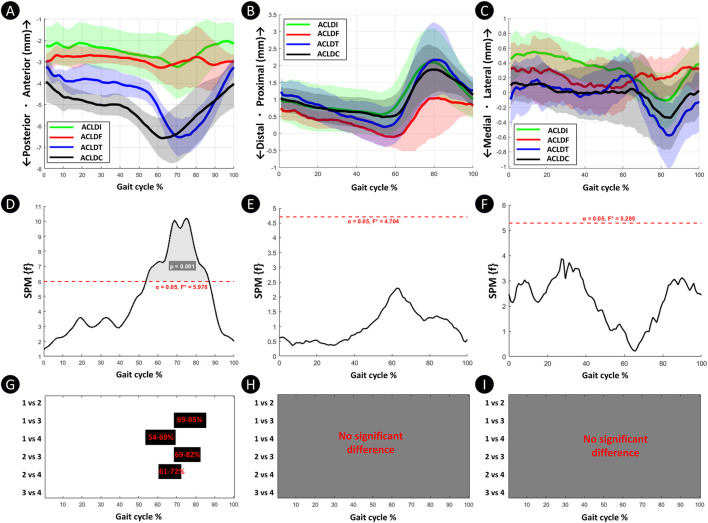
Comparison of knee joint kinematics among the four groups in the anterior-posterior, proximal-distal, and medial–lateral translation dimensions throughout the full gait cycle. Plots **(A–C)** Knee joint motion angles for the four groups in the anterior-posterior, proximal-distal, and medial–lateral translation dimensions; Plots **(D–F)** One-way SPM{F} analyses of intergroup differences across the three dimensions, with shaded regions indicating gait cycle intervals exhibiting statistically significant differences; Plots **(G–I)** Post hoc pairwise SPM{t} comparisons between groups for the anterior-posterior, proximal-distal, and medial–lateral translation dimensions, where black bars denote gait phases with significant between-group differences. 1 = ACLDI; 2 = ACLDF; 3 = ACLDT; 4 = ACLDC.

Figures 4A–C illustrate femoral–tibial displacements in the anterior–posterior, proximal–distal, and medial–lateral directions. No significant intergroup differences were detected in proximal–distal or medial–lateral translation. However, significant differences were observed in anterior–posterior displacement.

Post hoc comparisons (Figures 4G–I) showed that ACLDT and ACLDC had significantly greater anterior tibial translation than ACLDI. Compared with ACLDF, both groups also demonstrated increased anterior translation. These results indicate that tibial and combined femoral–tibial cartilage lesions are associated with excessive anterior tibial motion during gait.

### 3.3 Gait characteristic differences among the four groups

**FIGURE 5:**
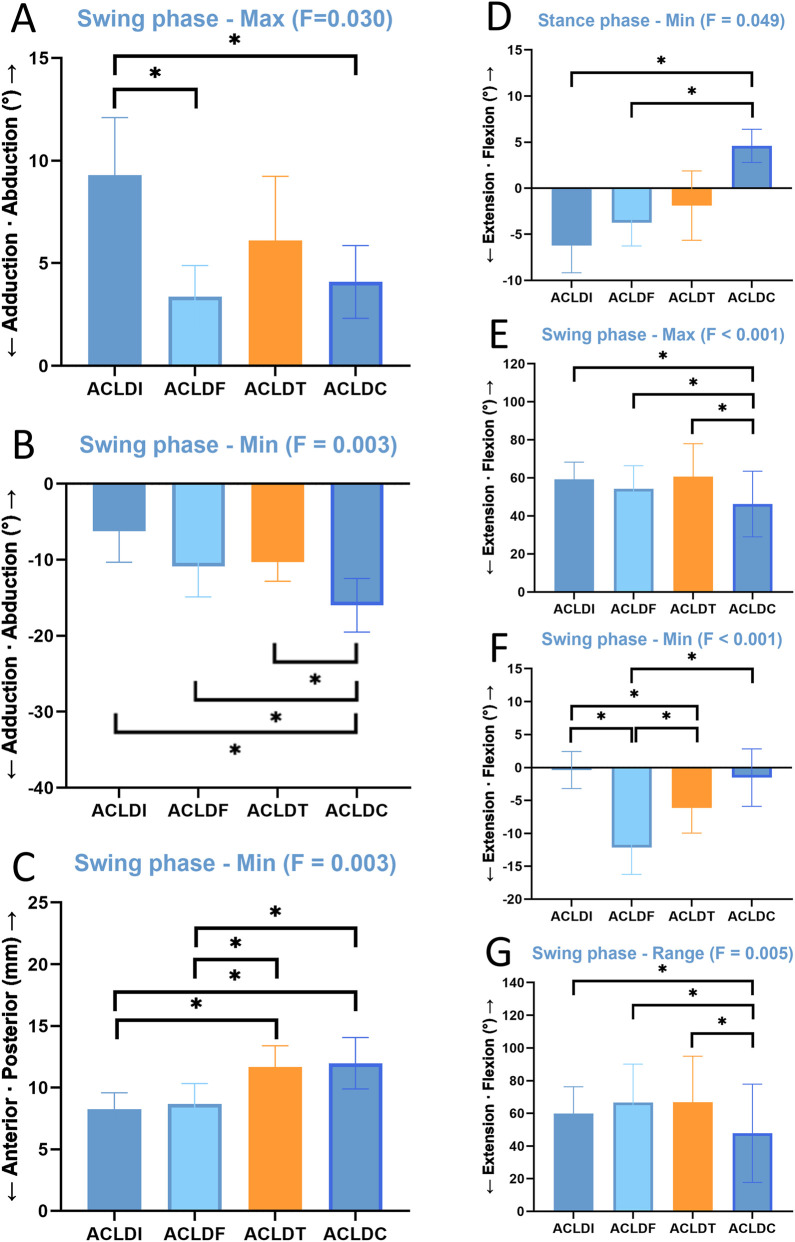
Covariate-adjusted group means for discrete gait parameters that remained significantly different among groups after adjustment for meniscal variables. **(A,B)**, swing-phase maximum and minimum values in the adduction–abduction dimension. **(C)**, swing-phase minimum value in the anterior–posterior translation dimension. **(D–G)**, flexion–extension parameters, including stance-phase minimum value **(D)**, swing-phase maximum value **(E)**, swing-phase minimum value **(F)**, and swing-phase range of motion **(G)**.

To further address the potential confounding effect of concomitant meniscal treatment, key discrete gait parameters were reanalyzed using covariate-adjusted linear models. Table 2 and Figure 5 summarize the parameters that remained significantly different among groups after adjustment for meniscal variables. Significant adjusted group effects were retained for selected adduction–abduction, flexion–extension, and anterior–posterior parameters.

#### 3.3.1 Adduction–abduction

Significant adjusted differences were observed in the swing-phase maximum and minimum values. ACLDI showed a greater swing-phase maximum value than ACLDF (p = 0.007) and ACLDC (p = 0.016). ACLDC showed a smaller swing-phase minimum value than ACLDI (p < 0.001) and ACLDT (p = 0.03).

#### 3.3.2 Flexion–extension

Significant adjusted group effects were found for the stance-phase minimum value and for the swing-phase maximum, minimum, and range values. ACLDC showed a greater stance-phase minimum value than ACLDI (p = 0.008) and ACLDF (p = 0.041), but a smaller swing-phase maximum value than ACLDI (p < 0.001), ACLDF (p = 0.018), and ACLDT (p < 0.001). ACLDF and ACLDT showed smaller swing-phase minimum values than ACLDI (p < 0.001 and p = 0.043, respectively). ACLDC also showed a smaller swing-phase range than ACLDT (p = 0.002) and ACLDF (p = 0.002).

#### 3.3.3 Anterior–posterior displacement

A significant adjusted group effect remained for the swing-phase minimum value. ACLDT and ACLDC showed greater anterior tibial displacement than ACLDI (p = 0.007 and p = 0.003, respectively) and ACLDF (p = 0.015 and p = 0.008, respectively).

## 4 Discussion

The principal finding of this study was that, in ACLD knees, cartilage lesion location was associated with distinct patterns of gait biomechanical abnormality. Between-group differences were primarily observed in abduction-adduction, flexion-extension, and anterior-posterior translation, and several key differences remained after adjustment for meniscal treatment. These findings suggest that concomitant cartilage injury in ACLD knees should not be regarded merely as an accompanying structural finding. Rather, cartilage lesion location itself may be an important determinant of gait functional phenotype.

Among the lesion patterns, combined femoral-tibial cartilage involvement showed the broadest gait kinematic abnormality. Compared with isolated ACL deficiency, the ACLDC group demonstrated greater coronal-plane deviation during swing, more restricted sagittal-plane motion, and abnormal anterior-posterior control. Previous studies have rarely stratified ACLD knees according to cartilage lesion location. However, Zeng et al. reported that specific walking kinematic patterns were associated with early cartilage lesion characteristics after ACL reconstruction, supporting a structure-function coupling between cartilage status and gait phenotype (Zeng et al., 2022). From a broader biomechanical perspective, Andriacchi et al. proposed that abnormal gait mechanics may alter contact location and load distribution, thereby affecting cartilage morphology and osteoarthritic progression (Andriacchi et al., 2009), whereas Haughom et al. linked abnormal tibiofemoral kinematics to early cartilage matrix degeneration detected by MRI (Haughom et al., 2012). Although these studies did not directly compare gait patterns across cartilage lesion locations in ACLD knees, they collectively support a potential bidirectional relationship between gait abnormality and cartilage status. Our findings extend this concept by suggesting that when both femoral and tibial articular surfaces are involved, gait abnormality becomes more multidimensional.

ACLDT was characterized more prominently by increased anterior tibial translation during walking. This suggests that, relative to isolated ACL deficiency, local abnormalities in support and contact behavior associated with tibial plateau cartilage damage may further impair anterior-posterior control during dynamic weightbearing. Lovejoy and Walker et al. emphasized that the tibial plateau serves not only as a principal contact-support surface during loading but also contributes to load distribution through maintenance of articular congruity and contact area (Lovejoy, 2007; Nordin, 2020).

In contrast, abnormalities in ACLDF were concentrated more in the sagittal-plane motion pattern, particularly reduced peak swing-phase flexion. This suggests that femoral-side cartilage damage may more directly disrupt femoral condyle rolling-sliding coordination on the tibial plateau, resulting in a stiffer gait strategy. Reinold and Vaienti et al. noted that normal knee flexion requires continuous rolling, sliding, and contact-point migration of the femoral condyles, and that this process depends heavily on normal articular morphology and the cartilage contact environment (Reinold et al., 2006; Vaienti et al., 2017; Zhou et al., 2021).

From a broader biomechanical and clinical perspective, these findings suggest that ACLD knees do not represent a single functional abnormal state but may instead comprise distinct residual instability subtypes. Accordingly, clinical management should move beyond the broad category of “ACL deficiency with concomitant cartilage injury” and instead consider the specific functional imbalance associated with lesion location. Patients with tibial lesions may require greater emphasis on dynamic anterior-posterior control, those with femoral lesions on restoring sagittal-plane excursion and addressing a stiffened gait pattern, and those with combined lesions on more comprehensive functional management. Thus, lesion-specific stratification may help guide more targeted rehabilitation and individualized management and may also have implications for long-term joint preservation (Rothrauff et al., 2022; Vannini et al., 2016).

Several limitations should be acknowledged. First, because this was a cross-sectional study, the present findings demonstrate associations between cartilage lesion location and gait functional phenotype but cannot establish causality or predict postoperative outcomes. However, because the aim of this study was to identify dynamic functional differences associated with lesion location, this design still provides direct descriptive evidence. Second, only 6 degrees-of-freedom knee kinematics were analyzed. Accordingly, the identified abnormalities should be interpreted primarily as knee-level functional phenotypes rather than as the complete compensatory strategy of the entire lower-extremity kinetic chain. Future studies should incorporate the hip, ankle, and trunk to better characterize whole-limb compensation. Finally, although meniscal treatment was adjusted for, other concomitant intra-articular pathologies may still have introduced residual confounding. Nevertheless, the persistence of key differences after adjustment suggests that the relationship between cartilage lesion location and gait abnormality is relatively robust.

## 5 Conclusion

In ACLD knees, gait biomechanical abnormalities are associated with cartilage lesion location. Different lesion locations correspond to distinct functional phenotypes, with tibial lesions more closely linked to anterior-posterior control deficits, femoral lesions to sagittal-plane motion restriction, and combined femoral-tibial lesions to more widespread multiplanar abnormality. These findings suggest that cartilage lesion location is not merely a structural descriptor but a biomechanically meaningful factor in functional assessment. Lesion-specific evaluation may improve identification of residual instability and support more individualized rehabilitation and joint-preservation strategies aimed at restoring stability and reducing secondary degeneration.

## Data Availability

The raw data supporting the conclusions of this article will be made available by the authors, upon reasonable request.
